# Rationally Targeted Mutations at the V1V2 Domain of the HIV-1 Envelope to Augment Virus Neutralization by Anti-V1V2 Monoclonal Antibodies

**DOI:** 10.1371/journal.pone.0141233

**Published:** 2015-10-22

**Authors:** Guomiao Shen, Chitra Upadhyay, Jing Zhang, Ruimin Pan, Susan Zolla-Pazner, Xiang-Peng Kong, Catarina E. Hioe

**Affiliations:** 1 Veterans Affairs New York Harbor Healthcare System – Manhattan Campus, New York, New York, United States of America; 2 Department of Pathology, New York University School of Medicine, New York, New York, United States of America; 3 Department of Biochemistry and Pharmacology, New York University School of Medicine, New York, New York, United States of America; Emory University, UNITED STATES

## Abstract

HIV-1 envelope glycoproteins (Env) are the only viral antigens present on the virus surface and serve as the key targets for virus-neutralizing antibodies. However, HIV-1 deploys multiple strategies to shield the vulnerable sites on its Env from neutralizing antibodies. The V1V2 domain located at the apex of the HIV-1 Env spike is known to encompass highly variable loops, but V1V2 also contains immunogenic conserved elements recognized by cross-reactive antibodies. This study evaluates human monoclonal antibodies (mAbs) against V2 epitopes which overlap with the conserved integrin α4β7-binding LDV/I motif, designated as the V2i (integrin) epitopes. We postulate that the V2i Abs have weak or no neutralizing activities because the V2i epitopes are often occluded from antibody recognition. To gain insights into the mechanisms of the V2i occlusion, we evaluated three elements at the distal end of the V1V2 domain shown in the structure of V2i epitope complexed with mAb 830A to be important for antibody recognition of the V2i epitope. Amino-acid substitutions at position 179 that restore the LDV/I motif had minimal effects on virus sensitivity to neutralization by most V2i mAbs. However, a charge change at position 153 in the V1 region significantly increased sensitivity of subtype C virus ZM109 to most V2i mAbs. Separately, a disulfide bond introduced to stabilize the hypervariable region of V2 loop also enhanced virus neutralization by some V2i mAbs, but the effects varied depending on the virus. These data demonstrate that multiple elements within the V1V2 domain act independently and in a virus-dependent fashion to govern the antibody recognition and accessibility of V2i epitopes, suggesting the need for multi-pronged strategies to counter the escape and the shielding mechanisms obstructing the V2i Abs from neutralizing HIV-1.

## Introduction

Vaccines are urgently needed to control HIV-1 infection worldwide, but the development of efficacious vaccines against HIV-1 remains an unsolved challenge. The RV144 prime-boost vaccine regimen tested in a phase III clinical trial in Thailand is the only candidate vaccine showing an efficacy that reaches 60% after 1 year but declines to ~30% after 3.5 years of follow up [1]. Although the immune correlates for the protection are not fully understood, the presence of higher titers of antibodies (Abs) against the V1V2 region of the HIV-1 envelope (Env) gp120 is associated with lower rates of HIV-1 acquisition among the vaccine recipients [1–5]. More recent studies in the SIV and macaque model recapitulated these findings [6–8], further supporting the potential functions of anti-V1V2 Abs in reducing the risk for HIV-1/SIV infection. Nonetheless, it remains unclear as to how these Abs exert their anti-viral activities to prevent virus infection [9].

A number of monoclonal antibodies (mAbs) against V1V2 have been isolated from HIV-infected individuals and from RV144 vaccine recipients [10–12]. Thus far, these mAbs have been categorized into at least three categories. The first group of mAbs is designated as V2q (quaternary) mAbs for mAbs such as PG9 and PG16 recognizing the quaternary epitopes that are presented preferentially on the virus Env trimers and encompass key N-glycans emanating from the V1V2 loop. PG9 and PG16 display potent neutralizing activities against 73–78% diverse HIV-1 isolates from different subtypes and circulating recombinant forms (CRFs) [10], but induction of such V2q Abs by vaccination is yet to be accomplished. Indeed, potent and broad virus-neutralizing activities were not induced in the RV144 vaccine recipients, and the detected virus neutralization did not correlate with reduced risk of HIV-1 acquisition [1, 9]. Two mAbs isolated from the RV144 vaccine recipients belong to the second category of V1V2 mAbs designated as V2p (peptide); these mAbs bind to V2 peptides from the region overlapping with the V2q epitopes but their binding and neutralizing activities are much more restricted than those of the V2q mAbs [11, 13]. The third category of V1V2 mAbs is defined by V2i (integrin) mAbs derived from HIV-1 infected individuals, V2i mAbsrecognize highly conformation-dependent conserved epitopes in the V1V2 region that encompass in part the integrin α4β7-binding motif [12, 14, 15]. Although the V2i mAbs are broadly reactive with a large array of gp120 proteins from multiple HIV-1 subtypes and circulating recombinant forms, these Abs do not have potent neutralizing activities against these viruses [12, 16]. Indeed, when tested in the standard neutralization assay with one hour of virus-mAb pre-incubation time, most of these mAbs are effective only against highly sensitive Tier 1 viruses and do not neutralize Tier 2 and Tier 3 viruses [12]. However, our recent studies demonstrate that these V2i mAbs are capable of neutralizing some of the relatively resistant Tier 2 viruses when the incubation time for mAb-virus interaction is prolonged up to 18 or 24 hrs [16]. Based on these findings, we postulate that although the V2i mAbs target conserved epitopes around the integrin α4β7-binding motif, the V2i epitopes are often shielded and are accessible or recognizable only momentarily. The mechanisms by which Abs are obstructed from recognizing these V2i epitopes are yet to be defined.

In this study we evaluated the specific elements in the distal V1V2 domain elucidated in the crystal structure of the V2i epitope bound by mAb 830A [17] and assessed by mutational analysis their importance in modulating the ability of the V2i mAbs to recognize the epitopes and exert HIV-1 neutralizing activities. The 830A epitope is at the distal end of the V1V2 domain on the opposite side from the PG9 epitope [17]. The key components of the 830A epitope include discontinuous amino acids at V2 positions 175, 177, and 179–180, which form a kink and overlap with the integrin-binding LDI/V motif, positions 153 and 154 in the B strand of V1, and position 194 at the C terminus of V2 [17]. In the crystal structures of the pre-fusion SOSIP Env trimers [18, 19], this area includes the unresolved hypervariable regions of V1 and V2 loops connecting A-B strands and C-D strands, respectively, and is partially shielded by V3. The mutational analyses conducted in this study demonstrate that the lack of the LDI/V motif in HIV-1 isolates such as 6535 and REJO did not account for their poor neutralization by V2i mAbs, as mutations restoring the motif have minimal effect on the activities of most V2i mAbs. In the case of the virus isolate ZM109, a charge change at position 153 from the unusual basic residue R to the more common acidic amino acid E greatly enhanced the virus sensitivity to neutralization by several V2i mAbs. Independently, the addition of a disulfide (S-S) bond to constrain the highly flexible V2 loop adjacent to the α4β7 integrin binding motif also improves neutralization of ZM109, BaL, and SF162 viruses by many of the V2i mAbs tested. These data indicate that, depending on the virus strains, the V2i epitopes may escape or be occluded from antibody recognition by different mechanisms, including single-point epitope mutations and conformational masking due to the structural flexibility of the V2i epitopes.

## Materials and Methods

### Cell lines, plasmids and viruses

Plasmids expressing HIV-1 Env of 6535 (clone 3 (SVPB5)), BaL (clone BaL.01), REJO (pREJO4541 clone 67 (SVPB16)), and ZM109 (ZM109F.PB4, SVPC13) were used for generating HIV-1 pseudoviruses. These plasmids were obtained from Drs. David Montefiori, Feng Gao, Ming Li, John Mascola, B.H. Hahn, J. F. Salazar-Gonzalez, C.A. Derdeyn and E. Hunter through the NIH AIDS Research and Reference Reagent Program (ARRRP). The plasmid bearing SF162 (pIRESSF162) *env* was constructed in our laboratory [20]. The 293T/17 cells were used to produce pseudoviruses and purchased from the American Type Culture Collection (ATCC). The TZM-bl cell line used as target cells in the neutralization assay was obtained from Dr. John C. Kappes, Dr. Xiaoyun Wu, and Tranzyme Inc. through the NIH ARRRP.

Pseudoviruses were produced by co-transfecting 293T/17 cells with HIV-1 *rev-* and *env-* expressing plasmids and the pNL4-3Δenv R-E- plasmid using the jetPEI transfection reagent (Polyplus-transfect SA). Supernatants were harvested after 48 hrs and clarified by centrifugation and 0.45 μm filtration. Some virus stocks were concentrated with a 100kDa Amicon filter (Millipore). Single-use aliquots were stored at −80°C. Virus titration was performed on TZM-bl cells with the Beta-Glo Assay System (Promega) as described previously [21]. Virus titration was performed for each virus stock prior to its use in the neutralization assays.

### Human monoclonal antibodies

Seven V2i mAbs, a control mAb 1418 (specific for human parvovirus B19), V3 crown mAbs (2219, 447-52D, 3074), and the CD4-binding site (CD4bs) mAbs 654-D and 1331 were used in this study and produced as described previously [22–26]. Both 654-D and 1331 represent poorly neutralizing CD4bs mAbs and were used interchangeably. In some experiments, more potent and broadly neutralizing V2q mAb PG9, CD4bs mAbs NIH45-46 and b12, and recombinant CD4-IgG2 protein were also tested. PG9 was purchased from Polymun Scientific Immunbiologische Forschung GmbH, Austria or obtained from Dr. Wayne Koff (International AIDS Vaccine Initiative). The mAbs NIH 45–46 and b12 was obtained from Dr. Pamela Bjorkman and Drs. Dennis Burton and Carlos Barbas through the NIH ARRRP. CD4-IgG2 was also obtained through the NIH ARRRP from Progenics Pharmaceuticals, Inc.

### Site directed mutagenesis

Single or double mutations were introduced in the HIV-1 Env V1V2 region using the QuikChange II XL Site-Directed Mutagenesis Kit (Stratagene, La Jolla, CA), according to the manufacturer's instructions. All mutant constructs were sequenced to verify that the correct amino acid changes were made.

### Crystal structural modeling

Structural modeling and analysis were carried out using ICM and figures were generated using PyMol (Schrödinger, LLC). The gp120 trimer structure data was obtained from the PDB access number 4TVP.

### Neutralization assay

Virus neutralization was measured using a β-galactosidase-based assay (Beta-Glo Assay System, Promega) with TZM-bl target cells [27]. Briefly, serial dilutions of mAbs were added to the virus in half-area 96-well plates (Costar) and incubated for the designated time period at 37°C. TZM-bl cells were then added along with DEAE-dextran (6.25μg/ml; Sigma). After incubation for 48 hrs, a luciferin-galactoside substrate (6-O-β-galactopyranosyl-luciferin) was added. The cleavage of the substrate by β-galactosidase generates luminescent signals measured in relative luminescence units (RLUs). Each test and control condition was tested in duplicate or triplicate. Assay controls included replicate wells of TZM-bl cells alone (cell control) and TZM-bl cells with virus alone (virus control). Percent neutralization was determined on the basis of virus control under the specific assay condition (e.g. 1 hr or 24 hr pre-incubation of virus without mAbs). The virus inputs were the diluted virus stocks yielding equivalent RLUs (typically ~100,000 RLUs) under the different assay conditions.

### gp120 binding assay

The relative binding affinity of mAbs to gp120s from 6535, REJO, ZM109 and its mutant pseudoviruses were measured by a sandwich ELISA. The ELISA plates were coated with sheep anti-gp120 Abs (D7324, 2 μg/ml; Aalto BioReagents, Dublin, Ireland), blocked with DMEM containing 10% FBS, and incubated with 1% Triton X100-treated pseudoviruses. Serially diluted mAbs (0.01–10μg/ml) were then added for 2 hrs, and the bound mAbs were detected with alkaline phosphatase-conjugated goat anti-human IgG Fc and p-nitrophenyl phosphate substrate. HIV+ serum pool obtained anonymously was used for normalization of the gp120 input and CD4-IgG2 binding was also assessed for control.

### Statistical analysis

Comparisons of virus neutralization and mAb binding were performed using GraphPad Prism 6. Statistical analyses were performed on neutralization data which reached ≥50%. Comparison of neutralization data was done on the mAb titration curves with a two-way ANOVA test.

## Results

**Fig 1:**
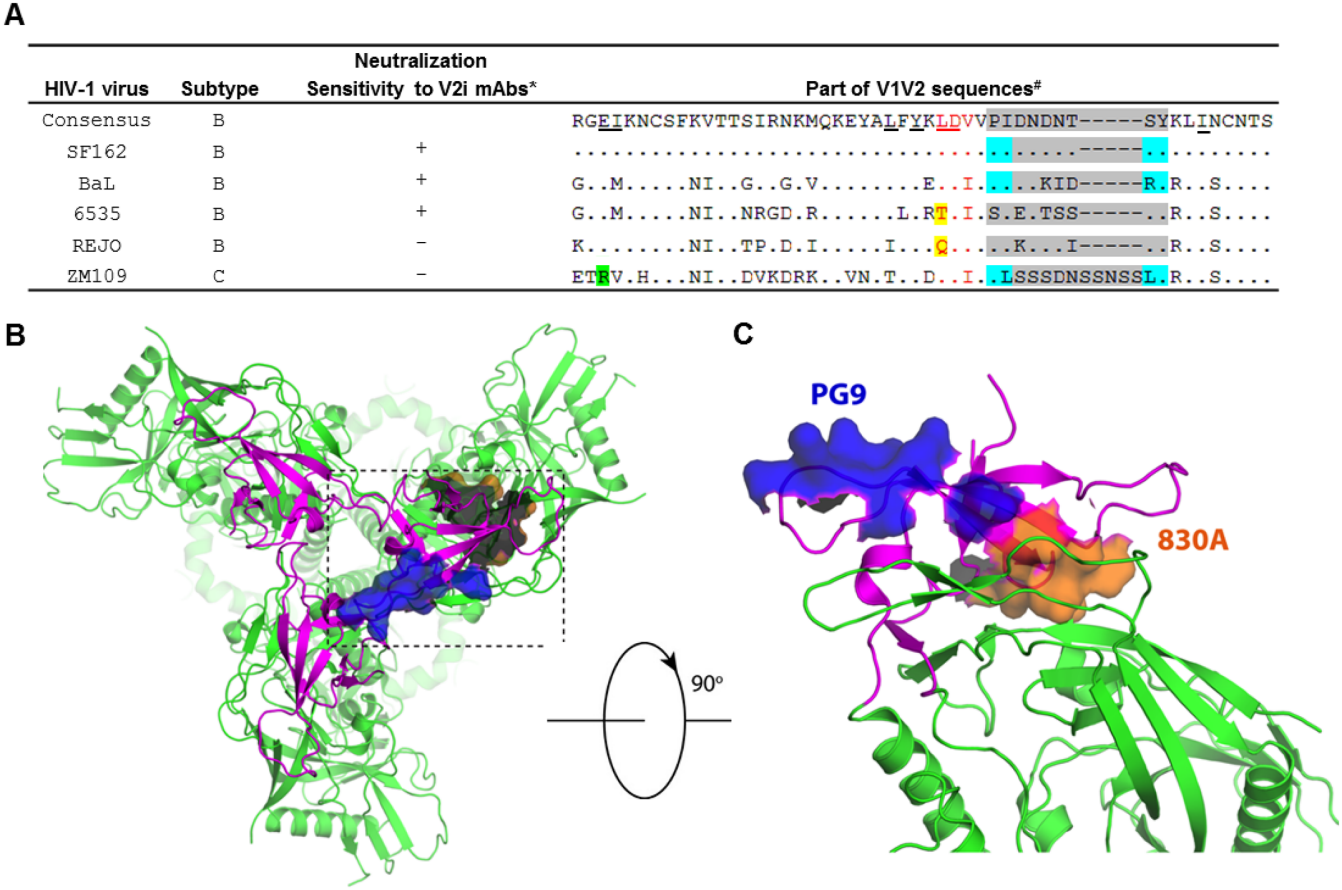
Alignment of V1V2 sequences from HIV-1 isolates tested and the HIV-1 Env trimeric structure with the 830A epitope. **(A)** Comparison of V1V2 sequences from HIV-1 isolates tested in this study. These five viruses display distinct neutralization sensitivity to V2i mAbs [12]. The key contact residues for mAb 830A are underlined. The integrin α4β7-binding motif ^179^LDV/I^181^ is shown in red, and amino acids that diverge from this motif are highlighted in yellow. The green highlight shows a distinct amino acid at position 153 in the V1 loop of the ZM109 strain. The gray highlight shows the sequence and length of the hypervariable V2 loop. To stabilize the flexible hypervariable V2 loop, disulfide bonds were introduced at positions highlighted in blue (183–191 and 184–190). **(B)** The surface of V2i mAb 830A epitope is illustrated on the pre-fusion Env trimer represented by the BG505 SOSIP structure (PDB Code 4TVP). The gp120 trimer is shown as ribbons and the V1V2 regions are colored in magenta. The 830A-binding site surface is colored in orange. However, since this surface faces down from this top view, only the majority of the backside (colored in dark grey) is seen. The surface of the PG9 epitope (colored in blue) is shown as a reference. **(C)** A zoom-in of the 830A epitope surface from an angle of view about 90 degree rotated from that in Fig 1B. For clarity, only one gp120 is shown.

This study sought to determine the elements within the distal region of the V1V2 domain that influence mAb recognition of the V2i epitopes in order to exert virus neutralization. To this end, HIV-1 pseudoviruses displaying varying degrees of neutralization sensitivity to V2i mAbs were selected (Fig 1A). The study focused on the distal V1V2 domain in which the V2i mAb 830A epitope is located and is at the opposite end from the epitope recognized by the V2q mAb PG9 (Fig 1B and 1C). We evaluated three components in this distal region for their contribution to Ab recognition of V2i epitopes by introducing specific mutations: 1) mutations at residue 179 that restore the ^179^LDV/I^181^ integrin-binding motif, 2) mutations at residue 153 that reinstate the highly conserved E in V1, and 3) introduction of a disulfide bond to stabilize the hypervariable region of V2 loop.

### Amino acid substitutions restoring the conserved integrin-binding LDV/I motif has minimal effects on virus neutralization by most V2i mAbs

**Fig 2:**
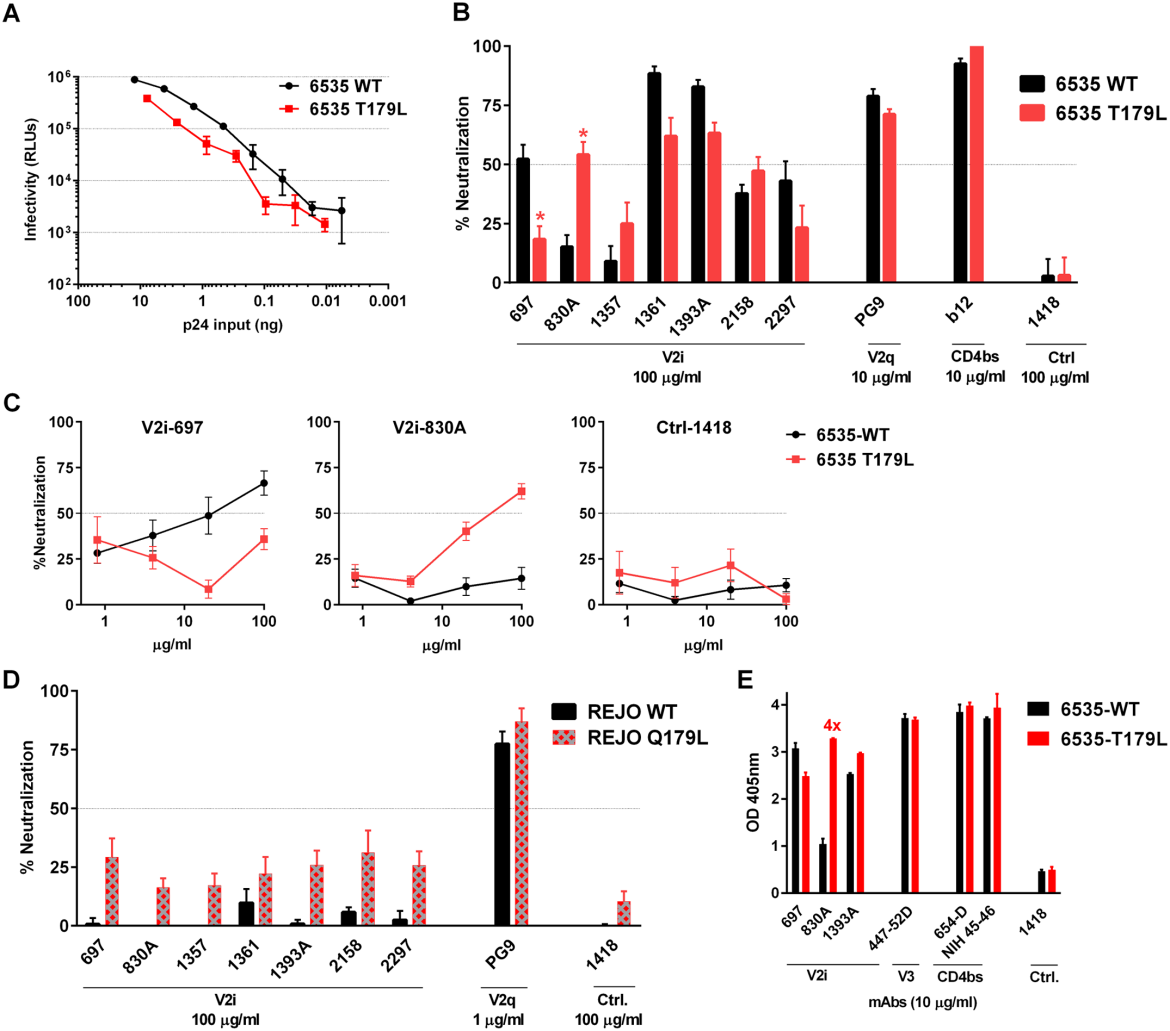
A single mutation creating the LDV motif has minimal effects on neutralization sensitivity of 6535 and REJO pseudoviruses to most V2i mAbs. **(A)** Infectivity of 6535 and its mutant T179L in the TZM-bl cells. Virus infectivity was measured based on β-galactosidase activity after 48 hrs. **(B)** Neutralization of 6535 and its mutant pseudoviruses by V2i, V2q, CD4bs, and irrelevant control mAbs. **(C)** Titration curves showing significant differences in neutralization of 6535 WT vs. T179L mutant by V2i mAbs 697 and 830A. *, p<0.05 based on two-way ANOVA test of the titration curves. **(D)** Neutralization of REJO WT and Q179L mutant pseudoviruses by V2i, V2q, and control mAbs. For neutralization assay, virus was pre-incubated with mAb for 1 hr at 37°C, and added to the TZM-bl cells. Inhibition of virus infectivity was measured by β-galactosidase activity. **(D)** ELISA reactivity of V2i, V3, CD4bs, and control mAbs to gp120 from 6535 WT and T179L mutant. For this assay, gp120s from virus lysates were captured onto 96-well plates by sheep anti-gp120 Abs, and reacted with mAbs tested. MAb binding was detected with alkaline phosphatase-conjugated goat anti-human IgG Fc and p-nitrophenyl phosphate substrate. Means and standard errors from two or more repeat experiments are shown.

An earlier mutagenesis study to map V2i epitopes revealed the importance of the α4β7 integrin binding motif for the reactivity of all seven human V2i mAbs tested [12, 14, 15]. Mutations in ^179^LDV/I^181^ motif, especially at positions 179 and 180 (HxB2 numbering) abrogates binding of most V2i mAbs to SF162 gp120. The x-ray crystallographic analyses of V2i mAb 830A in complex with its V1V2 epitope further showed residue ^179^L to be one of the key contacts for the 830A-V1V2 interaction [17]. Although the LDV/I motif is in general highly conserved among HIV-1 isolates [28], sequence comparison among the five HIV-1 strains studied here showed that two viruses, 6535 and REJO, have divergent amino acids at position 179: T and Q, respectively (Fig 1A). To determine whether these motif-altering substitutions contribute to the weak or no neutralizing activity of 830A and other V2i mAbs observed against these two viruses, T179L and Q179L mutations were introduced into the Env of 6353 and REJO, respectively, to restore the LDV/I motif. HIV-1 pseudotyped with the 6535 (T179L) Env retained its infectivity (Fig 2A), and became more sensitive to mAb 830A (Fig 2B). At the highest mAb concentration tested (100 μg/ml), neutralization of the mutant virus by 830A reached to >50%, whereas only 15% neutralization was observed with WT (Fig 2B). When 830A was titrated, a dose-dependent neutralization was observed with the 6535 (T179L) mutant but not with the WT counterpart (Fig 2C). Small increases were also observed with neutralization by mAbs 1357 and 2158, but the neutralizing levels remained below 50%. Significant changes were statistically determined for these and other neutralization data presented in this study when the titration curves reached 50%. No increase in virus neutralization was seen with V2i mAbs 697, 1361, 1393A, and 2297. The 6535 (T179L) mutation actually decreased neutralization by the V2i mAb 697 (Fig 2B and 2C). The 6535 (T179L) mutation also did not affect neutralization by the V2q mAb PG9 or the CD4bs mAb b12 (Fig 2B). In case of REJO (Q179L) (Fig 2D), all seven V2i mAbs showed varying low degrees of neutralization that were slightly higher than those against WT but remained <50% and were not significantly above that of the control mAb 1418. In comparison, high levels of neutralization were observed with V2q mAb PG9 against both REJO WT and Q179L mutant. Hence, the Q179L mutation did not markedly affect REJO sensitivity to V2i and V2q mAbs tested here.

To determine whether the changes observed with 6535 (T179L) neutralization by mAbs 830A and 697 are due to alterations in the mAb binding, ELISA was performed to test the binding activity of V2i mAbs to gp120 solubilized from the 6535 (T179L) mutant versus WT viruses. MAb 830A bound poorly to soluble gp120 from 6535 WT. The T179L mutation resulted in significant gain of 830A reactivity, and this correlated with enhanced neutralization of the 6535 (T179L) mutant by 830A. gp120 binding activities of V2i mAbs 697 and 1393A, V3 mAb 447-52D, the CD4bs mAbs 654-D and NIH45-46 were unchanged (Fig 2E). Hence, the 6535 (T179L) mutation improves gp120 binding and virus neutralization by mAb 830A but not by the other V2i mAbs. Overall, substituting the divergent amino acids at position 179 that creates the LDV/I motif specifically augments the interaction between 6535 and mAb 830A, but has minimal effects on REJO and the other V2i mAbs.

### A charge change at position 153 in V1 increases sensitivity of ZM109 to most V2i mAbs

**Fig 3:**
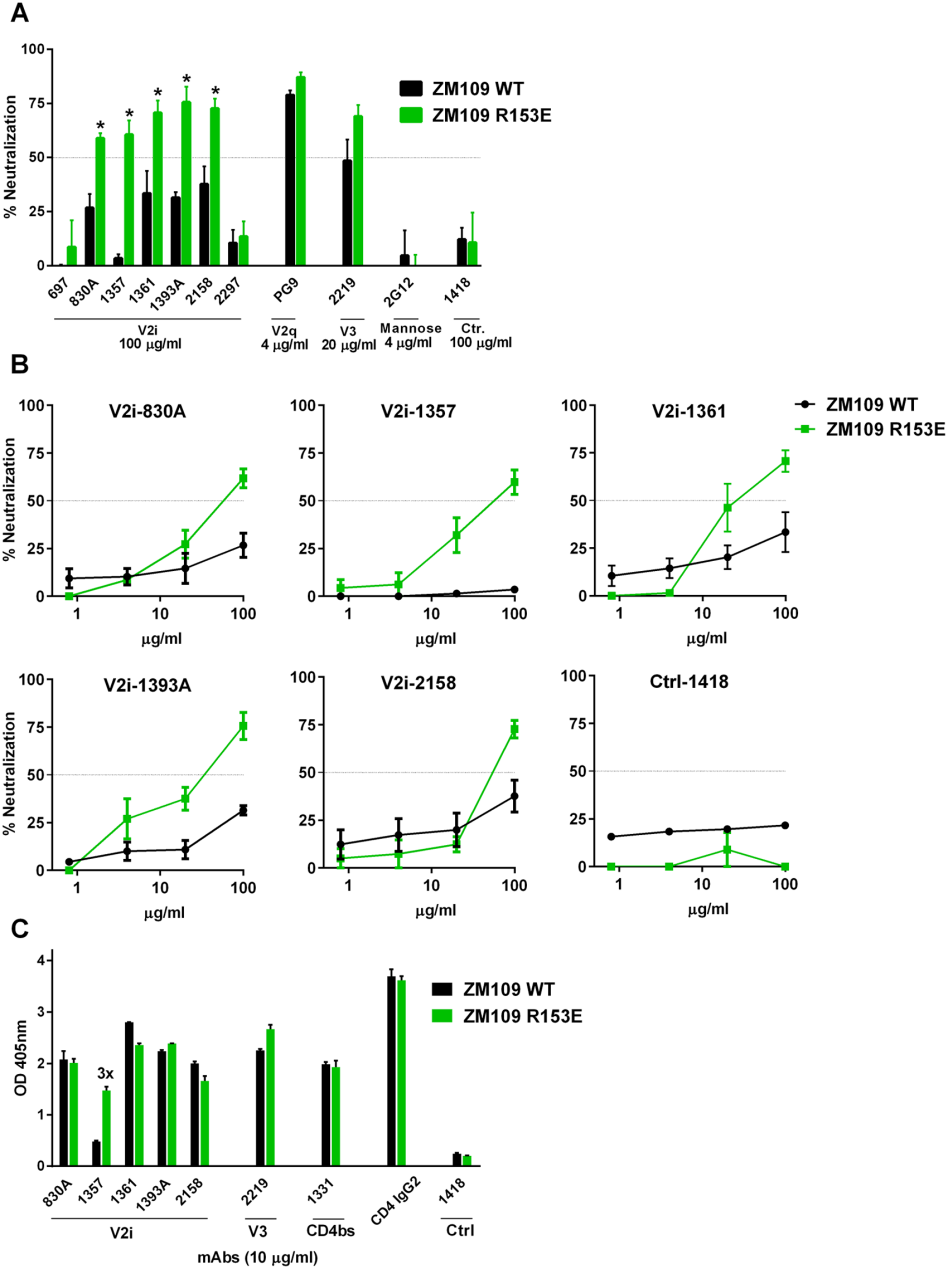
A charge change at position 153 in the V1 region of ZM109 increases the virus sensitivity to most V2i mAbs. **(A)** Neutralization of ZM109 WT and R153E mutant pseudoviruses by V2i and other mAbs. *, p<0.05 based on a two-way ANOVA test of the mAb titration results. **(B)** Titration curves showing differences in sensitivity of ZM109 WT vs. R153E to neutralization by five of the seven V2i mAbs. Statistical analyses were performed for neutralization data reaching above 50%. **(C)** ELISA reactivity of mAbs and CD4-IgG2 to gp120s from virus lysates of ZM109 WT and R153E.

Another key contact revealed by the crystallographic structure of the V2i mAb 830A in complex with its epitope is residue 153 in the V1 segment of the V1V2 domain. Although an acidic amino acid E is the most common residue at position 153 for subtype C and the vast majority of other HIV-1 subtypes [28], ZM109 virus (subtype C) has a basic residue R at this position (Fig 1A). When we made an R153E substitution, a dramatic increase was observed in the overall sensitivity of ZM109 to V2i mAbs, but not to V2q mAb PG9 and mannose-binding mAb 2G12 (Fig 3A). The slight increase seen with the V3 mAb 2219 also was not significant. Of the seven V2i mAbs tested, five showed significant increases in neutralization against the ZM109 (R153E) mutant virus as compared to WT. The neutralization was dose-dependent and reached >50% (Fig 3B). Interestingly, the mAb reactivity to solubilized gp120 was not markedly altered, except for V2i mAb 1357 (Fig 3C). mAb 1357 reacted poorly with gp120 of ZM109 WT, but bound better to gp120 of ZM109 (R153E), demonstrating that this mutation introduces to the ZM109 Env a more favorable epitope for mAb 1357. For the other V2i epitopes, the data suggest that the charge change at position 153 does not alter the epitopes themselves, but modulates the exposure of the epitopes when expressed in context of the functional virus-associated Env trimer. Nonetheless, the effects are specific to ZM109. The vast majority of HIV-1 isolates have the ^153^E residue on their Envs, but these viruses are resistant to neutralization by V2i mAbs (Fig 1A and [12, 28]), indicating the contribution of other elements in shielding V2i epitopes from Ab recognition.

### Stabilizing the hypervariable region of V2 loop by a disulfide bond enhances sensitivity to neutralization by V2i mAbs

**Fig 4:**
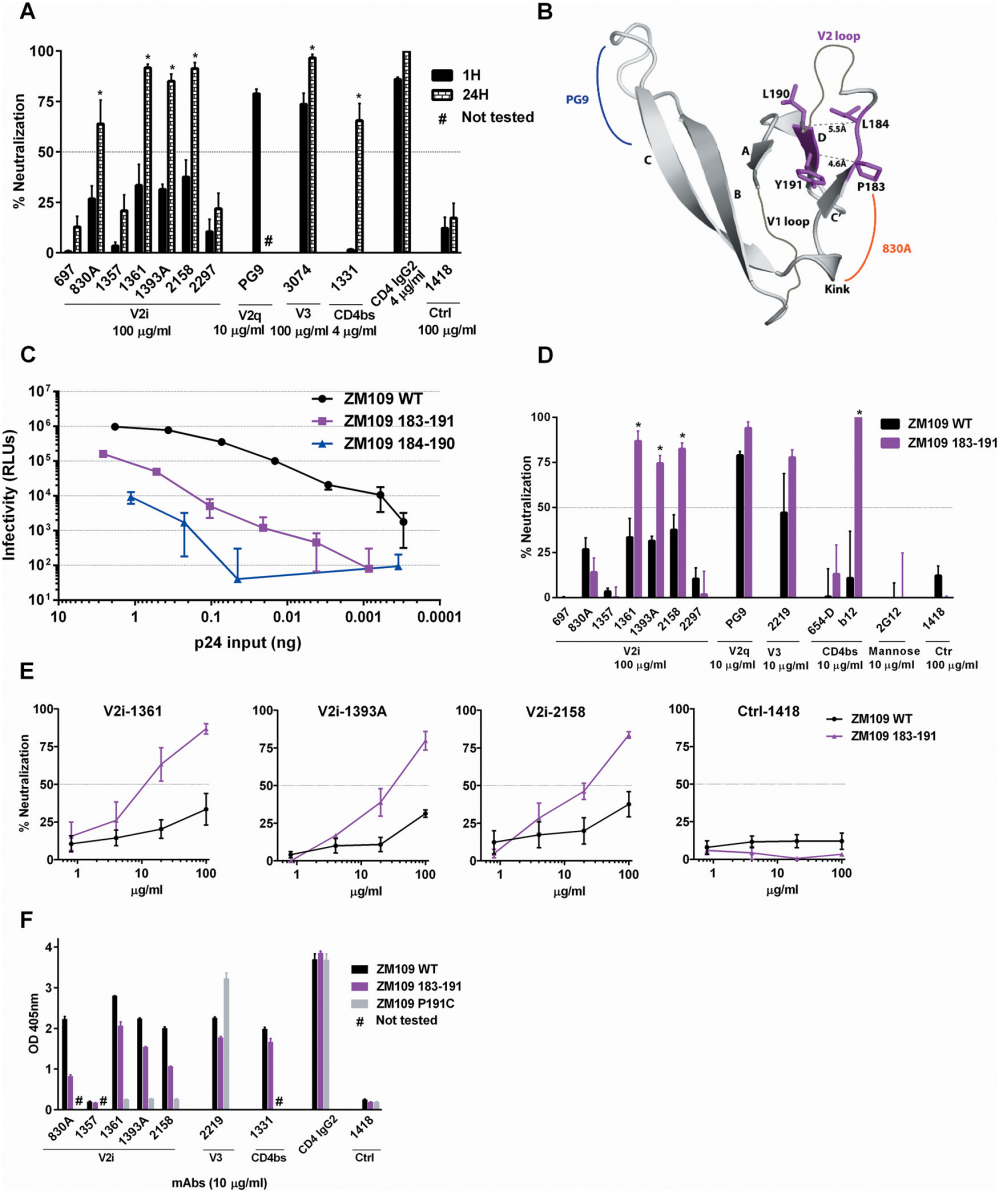
Addition of a disulfide bond to the flexible hypervariable V2 loop of ZM109 significantly increases the virus sensitivity to neutralization by some V2i mAbs. **(A)** Neutralization of ZM109 WT by V2i and other gp120-specific mAbs following virus-mAb pre-incubation for 1 hour vs. 24 hours. **(B)** Stabilization of the hypervariable region of V2 loop by a disulfide bond. The two disulfide bonds introduced individually were shown together in this model. The V1V2 region is rendered as ribbons with the mutated residues colored in magenta. The distances of the C atoms of the two pairs are indicated. The epitope locations of 830A and PG9 are indicated by orange and blue curves, respectively. **(C)** Infectivity of pseudoviruses with WT or mutant ZM109 Envs in TZM-bl cells. **(D)** Neutralization of ZM109 WT and the disulfide mutant (183–191) by V2i and other mAbs. **(E)** Titration curves showing differences in neutralization sensitivity of ZM109 WT and the 183–191 mutant to V2i mAbs 1361, 1393A, and 2158. Statistical analyses were performed for neutralization curves reaching above 50%. *, p<0.05 based on two way ANOVA test of the mAb titration curves. (**F**) ELISA reactivity of gp120s from ZM109 WT, 183–191, and the Y191C single mutant with V2i and other gp120-specific mAbs. CD4-IgG2 and the irrelevant mAb 1418 were also tested as controls.

Previous studies show that V2i epitopes are not readily accessible on most HIV-1 isolates for Ab recognition and a prolonged incubation time is required for the V2i mAbs to bind the virus Env and mediate neutralization [16]. Consistent with these findings, V2i mAbs 830A, 1361, 1393A and 2158 displayed significant neutralizing activity against ZM109 only after 24 hrs of virus-mAb pre-incubation (Fig 4A). Neutralization did not reach 50% with 1 hr of virus pre-incubation with each of the seven V2i mAbs, as reported earlier [12, 16]. The irrelevant control mAb 1418 had no neutralizing activity after 1 h or 24 hr incubation. The activity of the weakly neutralizing CD4bs mAb 1331 was also greatly improved by prolonging the pre-incubation, indicating that some CD4bs epitopes are masked similar to the V2i epitopes. On the other hand, V2q mAb PG9, V3 mAb 3074, and CD4-IgG2 readily neutralized ZM109 with 1 hr of virus-mAb pre-incubation, although 3074 was less potent requiring a 10-25x higher concentration to achieve the same level of neutralization, and its neutralization was further enhanced with 24 hrs of virus-mAb pre-incubation.

The failure of V2i mAbs to neutralize ZM109 within 1 hr of mAb treatment and their need for prolonged incubation suggests that often the V1V2 strands forming the V2i epitopes are inaccessible and/or do not adopt the conformations recognizable by the mAbs due to their structural flexibility. Notably, the epitope identified in the crystal structure of the 830A-V1V2 complex centers on a kink consisting of residues 175, 177, and 179–180 adjacent to the C-D strands connecting the hypervariable region of the V2 loop [17]. The C-D strands-connecting loop of ZM109, in particular, is much longer than those of other viruses (Fig 1A), which may render this region more flexible. To test this idea, we sought to stabilize the epitope by introducing a disulfide bond in the loop (Fig 4B). Two disulfide bonds were tested: one between residues 184 and 190 and another between residues 183 and 191. To create these disulfide bonds, C residues were introduced at the corresponding pairs to generate ZM109 Env with either L184C+L190C or P183C+Y191C substitutions. The ZM109 Env (184–190) pseudotyped virus was poorly infectious, whereas the pseudovirus with ZM109 Env (183–191) showed a reduced but sufficient level of infectivity for testing in the neutralization assay (Fig 4C). With only 1 hr of virus-mAb pre-incubation, ZM109 (183–191) was inhibited by three of the seven V2i mAbs (1361, 1393A, and 2158) in a dose-dependent way (Fig 4D and 4E). This neutralization pattern is reminiscent to that observed after 24 hrs of virus-mAb incubation (Fig 4A). These data indicate a possibility that the introduction of a disulfide bond to the V2 loop constrains the structural dynamics of the nearby V2i epitopes, enabling Abs to capture the epitopes and mediate neutralization in a shorter period of time. These effects were not seen with mAbs PG9 (V2q), 2G12 (mannose epitope), or 654-D (weakly neutralizing CD4bs), demonstrating that the mutations do not inherently alter the virus sensitivity to all Abs. However, virus sensitivity to the CD4bs mAb b12 was significantly enhanced and there was a trend towards greater sensitivity to the V3 mAb 2219 (Fig 4D), suggesting that the disulfide bond may also induce alterations in the Env configuration that render certain epitopes in the CD4bs and the V3 loop more accessible.

ELISA was then performed to assess whether the stabilizing effects on V2i epitopes of 1361, 1393A, and 2158 were apparent in the context of soluble gp120. Unexpectedly, the 183–191 mutations reduced ZM109 gp120 recognition by the three neutralizing V2i mAbs (Fig 3F). The mutations also nearly abrogated the reactivity of mAb 830A (Fig 3F), which correlated with the ineffectiveness of this mAb against ZM109 (183–191) (Fig 4D). The V2i mAb 1357 did not react with ZM109 gp120 and was unaffected by the 183–191 mutations. For comparison, we also tested V3 mAb 2219, CD4bs mAb 1331 and CD4-IgG2, and found that they bound to gp120 of ZM109 (183–191) as well as WT gp120. Hence, the stabilization of the V2i epitopes (1361, 1393A, and 2158) and the changes incurred to the V3 and CD4bs epitopes were not discernable in the Env gp120 subunits. Rather, the mutations likely affect these sites only in the context of Env trimers on the virus. In the soluble gp120 monomers, the V2i epitopes are fully exposed and readily form the structures recognizable by the mAbs; the addition of an S-S bond may actually constrain this region to result in poorer binding by the V2i mAbs 1361, 1393A, and 2158. For comparison, ZM109 Env was also generated with only a single Y191C substitution. The Y191C gp120 retained CD4-binding activity and V3 mAb reactivity, but this mutation completely abrogated V2i mAb recognition (Fig 4F) and virus infectivity (data not shown), indicative of the aberrant folding of the V1V2 domain as a result of the mutation.

**Fig 5:**
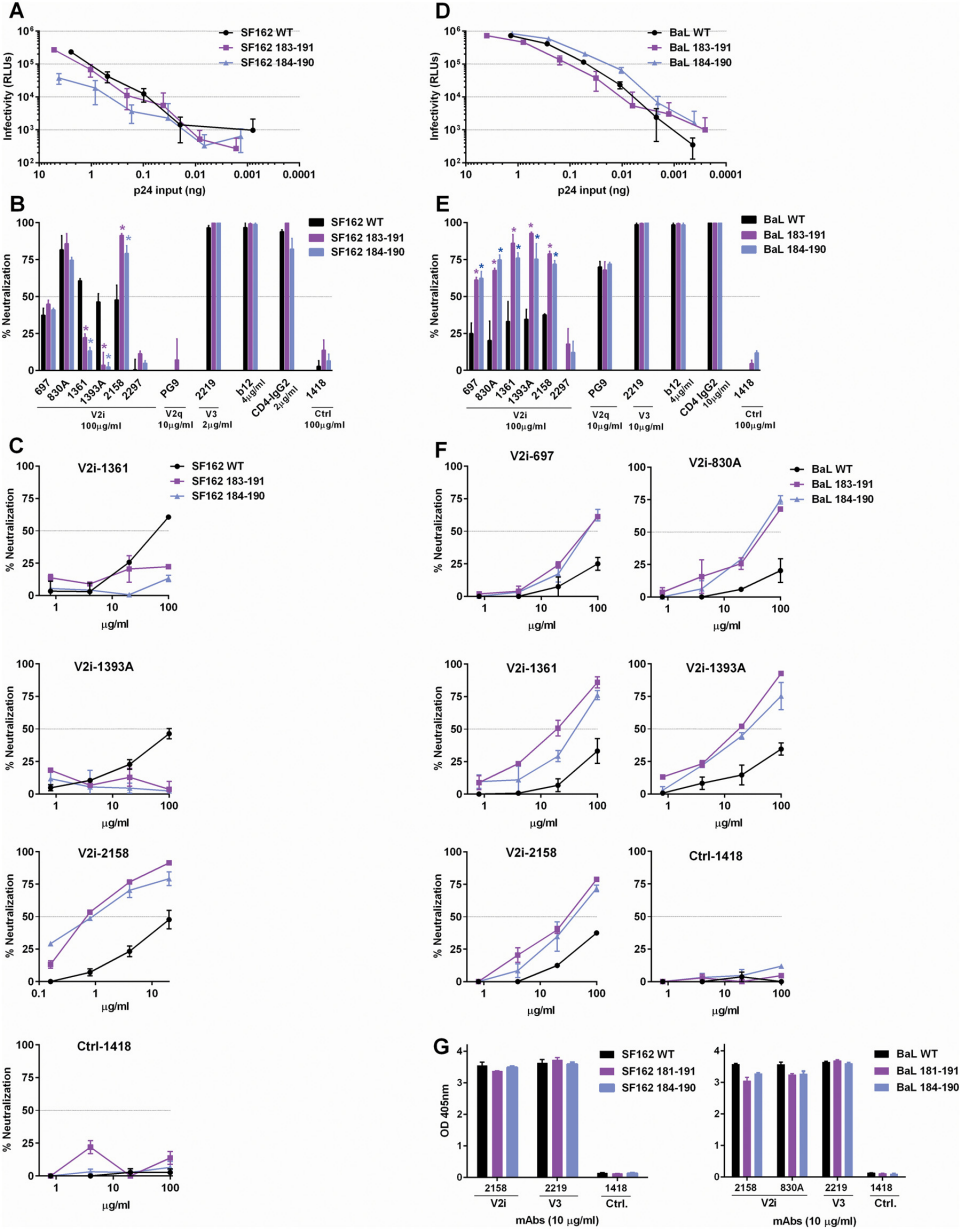
Addition of a disulfide bond to the hypervariable V2 loops of SF162 and BaL modulates virus sensitivity to V2i mAbs. **(A)** Infectivity of SF162 WT and its disulfide mutants in TZM-bl cells. **(B)** Neutralization of SF162 WT and its disulfide mutants by V2i and other mAbs. **(C)** Titration curves showing differences in neutralization sensitivity of SF162 WT and mutants to V2i mAbs 1361, 1393A, and 2158. **(D)** Infectivity of BaL.01 and its disulfide mutants in TZM-bl cells. **(E)** Neutralization of BaL.01 and its disulfide mutants by V2i and other mAbs. **(F)** Titration curves showing differences in neutralization sensitivity of BaL.01 WT and mutants to V2i mAbs 697, 830A, 1361, 1393A, and 2158. Statistical analyses were performed for neutralization curves reaching above 50%. *, p<0.05 based on two-way ANOVA test of the mAb titration data. **(G)** ELISA reactivity of V2i, V3, and control mAbs with gp120 from WT or S-S mutants of SF162 and BaL.01.

To evaluate whether the V2-stabilizing disulfide bond has similar effects on other viruses, we introduced pairs of C residues into the Env of SF162 and BaL.01 at positions 183 and 191 or positions 184 and 190 (HxB2 numbering). Viruses pseudotyped with the SF162 and BaL.01 mutants were infectious (Fig 5A and 5D), allowing us to test them in the neutralization assay with V2i and other mAbs (Fig 5B–5C and 5E–5F). As compared to WT, SF162 (183–191) and SF162 (184–190) were more sensitive to V2i mAb 2158 (Fig 5B and 5C). By contrast, the mutations reduced neutralization by V2i mAbs 1361 and 1393A, but these effects were observed only with SF162 and not with ZM109 (Fig 4) and BaL.01 (Fig 5E and 5F). The mutations did not alter neutralization of SF162 by V2i mAbs 697, 830A, and 2297, V3 mAb 2219, CD4bs mAb b12, and CD4-IgG2 (Fig 5B). The lack of neutralization by PG9 also remained unchanged. Hence, the addition of a disulfide bond to SF162 V2 increased or decreased virus sensitivity to three V2i mAbs but not the other mAbs tested. In contrast, BaL (183–191) and BaL (184–190) were neutralized better by all V2i mAbs tested, except 2297 (Fig 5E and 5F). Neutralization by V2q mAb PG9, V3 mAb 2219, CD4bs mAb b12, and CD4-IgG2 was unaffected. The mutations also did not affect the binding of V2i or V3 mAbs to the soluble gp120 subunits of SF162 or BaL.01 (Fig 5G). Taken together, these data show that the addition of a disulfide bond to the hypervariable region of V2 loop modulates the neutralization sensitivity of SF162 and BaL.01 to V2i mAbs without discernable impacts on mAb recognition of the monomeric Env subunits, but the effects vary depending on the individual mAbs and the virus strains.

## Discussion

This study sought to evaluate the elements within the distal end of the V1V2 domain of the HIV-1 Env for their contribution to the escape or shielding of the V2i epitopes from Ab recognition. Of the three elements studied here, amino acid substitutions at position 179 that restore the ^179^LDV/I^181^ motif have by and large minimal effects (Fig 2). In contrast a charge change at position 153 and the introduction of a S-S bond to the flexible V2 loop near the LDV/I motif show greater effects and enhance virus sensitivity to many of the V2i mAbs tested (Figs 3–5). The effects are most apparent on V2i mAbs, although the V3 crown and the CD4-binding site are also affected to varying degrees, particularly for the ZM109 virus. Nonetheless, no single alteration studied herein affect all V2i mAbs equally, indicative of the diversity of the epitopes targeted by these mAbs. The different V2i mAbs may recognize overlapping conformational-dependent epitopes involving the integrin-binding site in the V1V2 domain [12, 14, 15, 17], but each epitope is distinct. Likewise, the effects of the mutations evaluated here vary depending on the virus. Taken together, the data suggest that each V2i epitope is presented differently on the Env spikes of the individual virus isolates and that multiple mechanisms are used by the different viruses to escape from the neutralizing V2i Abs.

As observed with mAbs targeting the other Env epitopes [10, 29–35], single amino acid variation is also one of the strategies used by HIV-1 to evade neutralization by V2i mAbs, One example of this is the 6535 virus with a T179 residue which disrupts the key contact site for mAb 830A. A T179L substitution improved 6535 virus neutralization by the V2i mAb 830A, without similar effects on the other V2i mAbs (Fig 2B and 2C). ELISA data also showed enhanced binding specifically of 830A to gp120 of the 6535 T179L mutant (Fig 2E). These data are consistent with the structural data that ^179^L is one of the key contact points for 830A [17] and a single mutation at this residue is sufficient for evading mAb 830A. However, the 6535 T179L mutation has an opposing effect on mAb 697. The mutation reduced 6535 virus sensitivity to mAb 697, indicating the distinct epitopes targeted by mAbs 830A and 697. Moreover, since the activities of the other V2i mAbs were not altered, ^179^L is not critical for the other V2i epitopes as is for 830A. The importance of ^179^L residue is also virus dependent. An earlier study by Mayr et al. [15] showed that an L179D mutation disrupting the LDV/I motif in the SF162 Env drastically reduced the binding of 830A and three other V2i mAbs. In contrast, for the REJO virus with ^179^Q, the Q179L mutation did not affect 830A and all other V2i mAbs tested (Fig 2D). REJO also remains resistant to mAb 830A even after prolonged virus-mAb incubation ([16] and unpublished data). Hence, REJO utilizes additional escape or masking strategies yet to be defined to evade recognition by 830A.

Another example of a single amino acid variation that influences a V2i mAb is demonstrated by the ZM109 virus which is not recognized by the V2i mAb 1357 due to an unusual ^153^R residue (Fig 3). The ^153^R residue is another important contact site revealed in the crystal structures of mAb 830A and its epitope [17]. In most HIV-1 isolates of different subtypes, this position is occupied by an acidic E residue [28]. However, for the ZM109 virus, position 153 is occupied by a basic residue R and a complete charge change from R to E has a dramatic impact on ZM109 neutralization by many of the V2i mAbs; only 697 and 2297 were not affected and remained ineffective. The V2i mAb most affected by the ZM109 R153E mutation is mAb 1357 (Fig 3A and 3C). This mAb does not bind to the WT ZM109 gp120 and fails to neutralize ZM109 even with prolonged virus-mAb incubation time (Figs 3C and 4A). With the R153E substitution, the 1357 epitope is introduced to ZM109 Env, enabling the 1357 mAb to bind the ZM109 gp120 protein and to neutralize the virus. These data indicate that residue 153 is most likely an important contact site for the binding of mAb 1357 to its epitope. In contrast, V2i mAbs 697 and 2297 were not affected by the R153E mutation, demonstrating that the epitopes of these two mAbs are relatively independent of residue 153. Together with the results of the ^179^L mutations, these data support the idea that unique single-point mutations offer survival benefits for the different HIV-1 strains; however, this escape mechanism enables virus evasion from a particular Ab or a small subset of Abs, leaving the virus susceptible to many other Abs targeting even nearby overlapping epitopes on the HIV-1 Env.

In addition to epitope escape by amino acid variations, the V2i mAbs may be blocked from binding their epitopes and neutralizing the virus by epitope masking, without alterations on the epitopes themselves. Epitope masking has the potential to mediate a more pervasive impact, because multiple epitopes may be shielded simultaneously, enabling virus escape from a larger set of Abs. However, the epitopes are not perpetually masked. As shown by Munro et al. [36], the native unliganded Env trimers are structurally dynamic, capable of adopting multiple distinct configurations by spontaneously transitioning from a more stable “closed” conformation to at least two other less energetically stable conformations, which represent the “open” structures more accessible to Abs against often masked epitopes in the chemokine-receptor binding site as well as in the V1V2 and V3 loops. In this study, the ZM109 R153E mutation enhanced virus sensitivity to neutralization by four V2i mAbs (830A, 1361, 1393A, and 2158), even though each mAb binding to the ZM109 gp120 Env subunit was not changed (Fig 3). These data indicate that the mutation does not affect these four V2i epitopes directly; rather, it prods the Env spike toward the “open” configurations that render the V2i region more accessible to the mAbs. Previously, an E153G change has also been attributed to increased macrophage tropism for a number of viruses, while the reciprocal G153E reduced virus infectivity in macrophages [37]. Interestingly, residue 153 is spatially close to residue 178, which is a D in the ZM109 sequence. Thus, introducing another negative charge at position 153 may induce local distortions of the structure that can affect the trimer packing. This in turn can lead to the opening of the V1V2 region to make it more accessible. However, this scenario is pertinent only to ZM109. For other HIV-1 envelopes, such as BG505, a highly conserved E residue occupies position 153. This residue is spatially close to the side chains of an R at position 419 in the β19 strand and of two other Rs at positions 151 and 178 in the V1V2 domain [18, 38]. These electrostatic interactions likely contribute the stable closed Env conformation which buries a significant portion of the V1V2 surface, making the V2i epitopes in those virus strains poorly accessible.

Independent of the R153E mutation, the addition of a disulfide bond to the highly flexible V2 loop also augmented ZM109 sensitivity to neutralization by the V2i mAbs (Fig 4). A pair of substitutions to C residues at this loop rendered ZM109 susceptible to neutralization by three of the seven V2i mAbs (1361, 1393A and 2158). In the case of SF162, the extra disulfide bond augmented neutralizing activity of only one V2i mAb (2158) and reduced neutralizing activity of two of the V2i mAbs (Fig 5). Yet, the disulfide bond enhanced neutralization sensitivity of BaL.01 to all of the V2i mAbs except 2297 (Fig 5). Virus-specific effects were observed with individual V2 mAbs such as 1361 and 1393A: the addition of a disulfide bond improved neutralization of ZM109 and BaL.01 by both mAbs, but decreased neutralization of SF162. In contrast, the disulfide mutations enhanced neutralization of all three viruses by mAb 2158. These data indicate that the addition of the disulfide bond has varying effects that depend greatly on the particular virus-mAb pairs. However, in general SF162 is more refractory than BaL.01 and ZM109. Notably, the extra disulfide bond introduced to the V2 loop at the distal end of the V1V2 domain affected mainly the nearby V2i epitopes and had minimal effects on the PG9 (V2q) epitope at the opposing end of V1V2 domain. The V2i epitopes are made in part of the surface-exposed hypervariable V2 loop that is not concealed by other structures in the trimeric Env spike. The high flexibility of the loop enables the virus to avoid Ab recognition, as this loop segment may rarely adopt the particular conformations recognizable by the Abs. When stabilized as shown here by a disulfide bond, many of the V2i mAbs become more effective in neutralizing the virus. Nonetheless, the disulfide bond also impacted some epitopes in the V3 crown and the CD4 binding site, although the effects were apparent mainly on the more resistant virus ZM109 rather than on the sensitive viruses SF162 and BaL.01 considered to have more open Env configurations. These results suggest that, for ZM109, the addition of a disulfide bond to the V2 loop not only stabilizes this flexible region to favor the formation of V2i epitopes, but also exerts more global influence on the virus Env spike, altering the exposure of V2i epitopes as well as some epitopes n the V3 crown and the CD4-binding site.

The epitope masking has been extensively studied in the context of neutralizing epitopes at the crown of the V3 loop [16, 39–43], which together with the V1V2 domain form the apex of the HIV-1 Env spike. By contrast, V2i epitope masking has not been studied as much. Our recent data indicate that the masking for the V2i and V3 crown epitopes is mediated by distinct mechanisms [16]. The V3 crown is occluded by the V1V2 domain; this is evident in the pre-fusion BG505 Env structure where V1V2 covers most of the V3 loop especially its crown region [18, 38]. It is consistent with earlier studies showing that the V1V2 deletion renders some viruses more susceptible to V3 mAbs [44, 45]. The V3 crown also becomes more exposed after CD4 binds to the Env [46]. Further, most epitopes in the V3 crown can be affected by changes in N-linked glycans as a result of either treatment with mannosidase inhibitors or mutations at the glycosylation sites [16, 39, 47–51]. The V2i epitopes, on the other hand, are not affected by CD4 binding and are insensitive to mannosidase inhibitors. In this study, the introduction of a disulfide bond to the flexible V2 loop rendered the virus more sensitive to neutralization by many V2i mAbs, but had less dramatic effects on the V3-crown mAbs (Figs 4 and 5). Similarly, the ZM109 R153E mutation enhanced neutralization by most of the V2i mAbs, without affecting the V3-crown mAb to the same extent (Fig 3). Taken together, this study demonstrates that multiple virus-specific mechanisms are exploited to evade neutralizing Abs against the V2i and V3 crown epitopes, and also against some epitopes in the CD4-binding site. Due to the distinct mechanisms or factors involved in epitope escape and masking in these regions, simultaneous evasion from neutralizing Abs targeting V2i, V3, and the CD4bs may not be as readily achieved and may incur more fitness costs than thwarting Abs against only one of the sites. While further studies are undoubtedly needed to corroborate this notion, the findings of this study support a concurrent utilization or elicitation of neutralizing Abs targeting multiple disparate sites on the virus Env as one of the prerequisites to overcoming the diverse epitope escape and masking strategies deployed by HIV-1.
